# Health Education Campaign to Improve Malaria Knowledge, Prevention, and Treatment Behaviors in Rural East Nusa Tenggara Province, Indonesia: Protocol for a Cluster-Assigned Quasi-Experimental Study

**DOI:** 10.2196/66982

**Published:** 2025-05-01

**Authors:** Robertus Dole Guntur, Maria Lobo, Dony Martinus Sihotang, Yulianti Paula Bria, Damai Kusumaningrum

**Affiliations:** 1 Faculty of Science and Engineering Nusa Cendana University Kupang NTT Indonesia; 2 Faculty of Engineering Widya Mandira Catholic University Kupang NTT Indonesia; 3 Department of Husbandry State Agricultural Polytechnic Kupang NTT Indonesia

**Keywords:** malaria awareness, local wisdom-based malaria education campaign, rural community, malaria elimination, malaria health policy, COVID-19, malaria knowledge, malaria prevention measure knowledge and practice, malaria treatment-seeking behavior

## Abstract

**Background:**

Malaria is a major health issue that is distributed across 85 countries globally including Indonesia. Indonesia is in the process of achieving malaria elimination. Currently, a high burden of malaria exists in the rural eastern part of the nation, including East Nusa Tenggara Province where the number of malaria cases increased significantly during COVID-19. To achieve malaria elimination, malaria awareness must be measurable and integrated into malaria policy. Currently, malaria awareness among rural communities in the region is low, and interventional studies aiming at improving malaria awareness in rural areas in Indonesia are poorly documented.

**Objective:**

This study aims to investigate the impact of a local wisdom-based health education campaign combining local music, the voice of subdistrict leaders, and loudspeaker announcements on malaria-related behaviors in rural communities. Specifically, we aim to assess the effect of this intervention on (1) improvement in the malaria awareness index among rural communities and their associated factors, (2) changes in appropriate malaria treatment-seeking behavior (AMTSB) and its associated factors, (3) enhancements in knowledge and practice of malaria prevention measures and their associated factors, and (4) increased use of long-lasting insecticide-treated nets and their associated factors.

**Methods:**

This study used a cluster-assigned quasi-experimental design with pretest and posttest assessments in control and intervention groups. The control group, consisting of 12 villages, received malaria education integrated into routine health services provided by local health centers. The intervention group, comprising 13 villages, received the same education as the control group, in addition to a malaria campaign conducted once a week for 20 weeks. Before and after the campaign, a household survey was conducted to assess behavioral aspects of malaria, including general knowledge of malaria, AMTSB, and malaria prevention measures knowledge and practice. Improvement in the malaria awareness index, AMTSB, good level of malaria prevention measure knowledge, and good level of malaria prevention measure practice will be determined based on the difference scores for each index before and after the intervention in both groups. The chi-square test will be used to assess score differences. Binary logistic regression analysis will be conducted to identify key risk factors associated with changes in each index.

**Results:**

The intervention was conducted from the last week of August 2024 to the second week of January 2025. A total of 894 respondents participated before and after the intervention. The project is currently in progress, with multiple papers being drafted for publication in peer-reviewed journals.

**Conclusions:**

This study is expected to provide significant findings to comprehensively investigate the change in behavioral aspects of malaria due to a local wisdom-based malaria education campaign. The findings could assist stakeholders in Indonesia with developing malaria health policies that are contextually relevant, thereby supporting global efforts to achieve malaria-free status by 2030.

**International Registered Report Identifier (IRRID):**

DERR1-10.2196/66982

## Introduction

Malaria is a major health problem spreading across 85 countries globally, with an estimated 249 million people with malaria in 2022, of which 2% were from the Southeast Asia (SEA) region [1]. The action plan in this region indicates that the entire SEA region is targeted to achieve malaria elimination by 2030 [2]. Two countries in the region, Maldives and Sri Lanka, obtained a malaria elimination certificate from the World Health Organization (WHO) in 2015 and 2016, respectively, while other countries have a high burden of malaria, with India and Indonesia in particular contributing to 88.1% to the total number of malaria cases in the region [1]. One important indicator for a region to be categorized as having eliminated malaria is the absence of local malaria transmission cases for 3 consecutive years [3].

Following the global commitment, Indonesia has also set a national goal to eliminate malaria by 2030 [4]. Much progress has been obtained toward this commitment. Of the total 514 districts, 75.7% have received malaria elimination certificates since 2023 [5], and malaria is currently concentrated in rural areas [4]. However, there is a huge disparity between the western and eastern parts of the country in this achievement. Most of the districts in the western part have been classified as malaria elimination zones, while a limited number of districts have received malaria elimination certificates in eastern Indonesia, including some in East Nusa Tenggara (ENT) Province [5]. ENT Province has 22 districts and is the third largest contributor to malaria cases at the national level [6]. The number of malaria cases increased during the COVID-19 pandemic, from 12,909 in 2019 to 15,830 in 2022 [7]. Of the total number of districts in this province, 9 districts (41%) have received malaria elimination certificates, while the rest are still categorized as high, medium, or low endemic malaria areas [8]. East Sumba District is one of the high malaria-endemic settings in ENT Province, with the total number of malaria cases reaching 5537 in 2022 [9] and the majority of cases due to *Plasmodium falciparum* [10].

Malaria is a complex health issue influenced by multiple factors including *Plasmodium* types [11-13], sociodemographic factors [14-16], environmental factors [17-19], and behavioral aspects of malaria [20]. The distribution of malaria cases shows the different patterns within and between regions, due to variations in the environment, vectors, and social conditions of the local community [21]. Various studies have been conducted in ENT Province as an effort to reduce the incidence of malaria in this area [22-27]. Studies of the aspects of malaria parasites show that the prevalence of *P. vivax* malaria is greater than *P. falciparum* malaria [22-24], and this presents a major challenge for efforts to achieve malaria elimination considering that *P. vivax* malaria treatment requires high public awareness to complete the treatment for 14 days for the optimal results [28]. Studies on malaria vectors in this region show that the prevalence of mosquito biting in this area peaks at midnight both indoors and outdoors [25,26]. Studies of environmental aspects show that increasing the distance of residence from mosquito larval habitats is followed by a decrease of the risk of malaria in all age groups [27]. However, studies on the behavioral aspects of local communities are very poorly documented in ENT Province, though malaria knowledge significantly contributes to achieving malaria elimination [29,30]. Community behavior and their perspectives are very important in the efforts to achieve malaria elimination in certain areas [31-33].

In the context of Indonesia, several studies of community behavior regarding the knowledge and practice of malaria prevention have been conducted in various provinces [34-36]. The results of these studies indicate that community members have the awareness to practice several malaria prevention methods. However, all these observational studies were conducted in the western part of Indonesia where most of the districts have been categorized as malaria elimination zones [6].

Some studies on community behavior regarding the knowledge and practice of malaria prevention have been conducted in ENT Province [37-43]. A study conducted by the National Health Research Agency in 2018 in eastern Indonesia including ENT Province reported significant variations between provinces in terms of malaria prevention practices in rural communities [37]. Other studies on malaria prevention measures knowledge (MPMK) and malaria prevention measures practice (MPMP) in the region indicated that the Tetun ethnic group used traditional plants to prevent malaria [38], the use of mosquito nets was only common in rural communities of low endemic settings [43], and communities in high endemic areas have a low level of MPMK compared with community groups in low endemic areas [44]. Another study of malaria prevention measures revealed that knowledge on long-lasting insecticide-treated nets (LLINs), which are the most effective prevention measures to prevent malaria [33,45], was only common in the high endemic settings in this province [42]. Regarding malaria treatment-seeking behaviors of the local community, the current literature reveals that the prevalence of appropriate malaria treatment-seeking behavior (AMTSB) among rural communities in the province is very low [41]. Research on malaria awareness in this rural community demonstrated that the level of malaria awareness of rural communities in ENT Province was very low [39] and significant variation in malaria awareness was present among ethnic groups, with the Sumba ethnic group having the lowest level [40]. However, they were all observational studies that only observed the behaviors of rural communities at a certain time. The results of experimental studies on the behavioral aspects related to malaria in rural communities in ENT Province are less documented. This indicates that there have been no efforts by various parties to increase malaria awareness in rural communities in this region. Therefore, innovation is needed to increase malaria awareness among rural-dwelling adults who are an important community group to achieve malaria elimination [46]. Increasing malaria awareness in rural communities allows them to seek the right treatment if they are sick [33,47], reduces the prevalence of malaria [48], and accelerates the elimination of malaria [49].

In the global context, the literature indicates that interventional studies have significantly improved malaria knowledge [50,51], encouraged communities to adopt various malaria prevention measures [51-55], and promoted early malaria treatment-seeking behaviors [56]. In Indonesia, experimental studies using video media have substantially increased malaria knowledge among the elderly in Mimika District, Central Papua Province [57], and Kulon Progo District, Special Region of Yogyakarta [58]. However, these studies were conducted with small sample sizes. A current systematic review on interventional studies for malaria and dengue control indicates that the use of various media in interventions has enhanced disease knowledge among both the general population [59] and school communities [60]. However, there is a lack of studies in the Indonesia context focusing on this approach [59].

The use of loudspeaker media [61], use of local-based songs [62], and local government involvement [63] have been identified as highly effective for increasing health awareness among rural communities [61,62]. However, the impact of these three culturally relevant approaches on malaria-related behavioral changes in rural communities in Indonesia remains unexplored, despite rural populations contributing significantly to the country’s malaria burden [4]. Therefore, these interventional studies will fill these gaps with the following objectives: (1) determine the impact of locally based interventions on improving the malaria awareness index among rural communities and their associated factors including sociodemographic factors and environmental variables, (2) investigate the effectiveness of locally based interventions on improving AMTSB among rural communities and their associated factors, (3) assess the influence of a locally based intervention on enhancing knowledge and practice of malaria prevention measures among rural communities and their associated factors, and (4) estimate the effect of a locally based intervention on increasing the use of LLINs in rural settings and their associated factors.

This study is expected to provide significant findings to comprehensively clarify the change in the behavioral aspects of malaria in the rural ENT Province because of the application of a locally based intervention. Improvement in the malaria awareness index, variation in MPMK and MPMP, and a change in malaria treatment-seeking behaviors among rural communities will be recognized. The findings of this study could assist public health policy makers in Indonesia to design malaria policy to boost malaria elimination progress in the country. The findings might also be beneficial for other countries with similar sociodemographic characteristics to support global actions to achieve a malaria-free area by 2030.

## Methods

### Study Population

**Figure 1:**
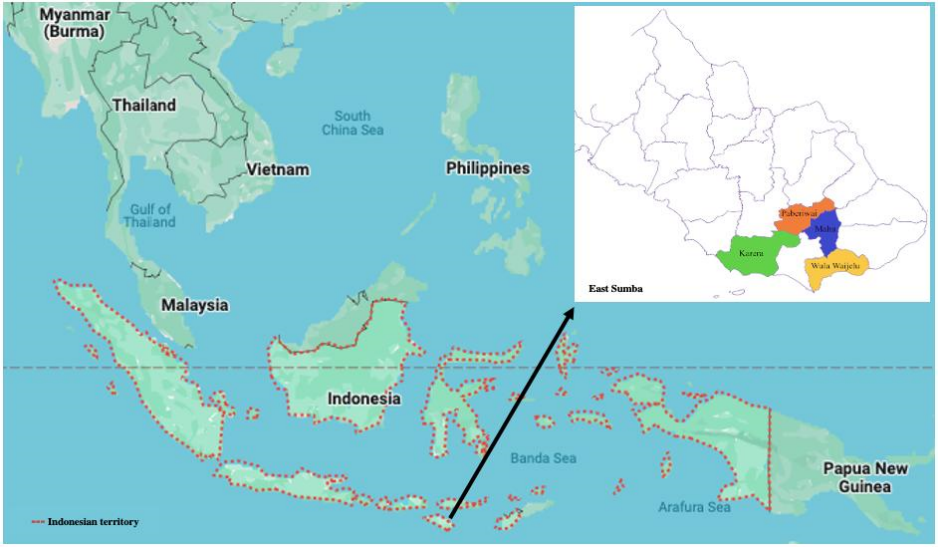
Map of study sites.

East Sumba District was selected as the study area because it is classified as a high malaria-endemic region [64]. The district consists of 22 subdistricts and 156 villages [65]. The total population in the district is 248,776, with an almost equal distribution of male (51.4%) and female (48.6%) residents. Approximately one-half of the population is engaged in the agricultural sector. The district has 4 hospitals, 22 public health centers, and 78 subsidiary public health centers across the region [65]. This study was conducted in 4 selected subdistricts where there is 1 public health center in each subdistrict, as indicated in Figure 1.

### Study Design and Sampling Technique

**Figure 2:**
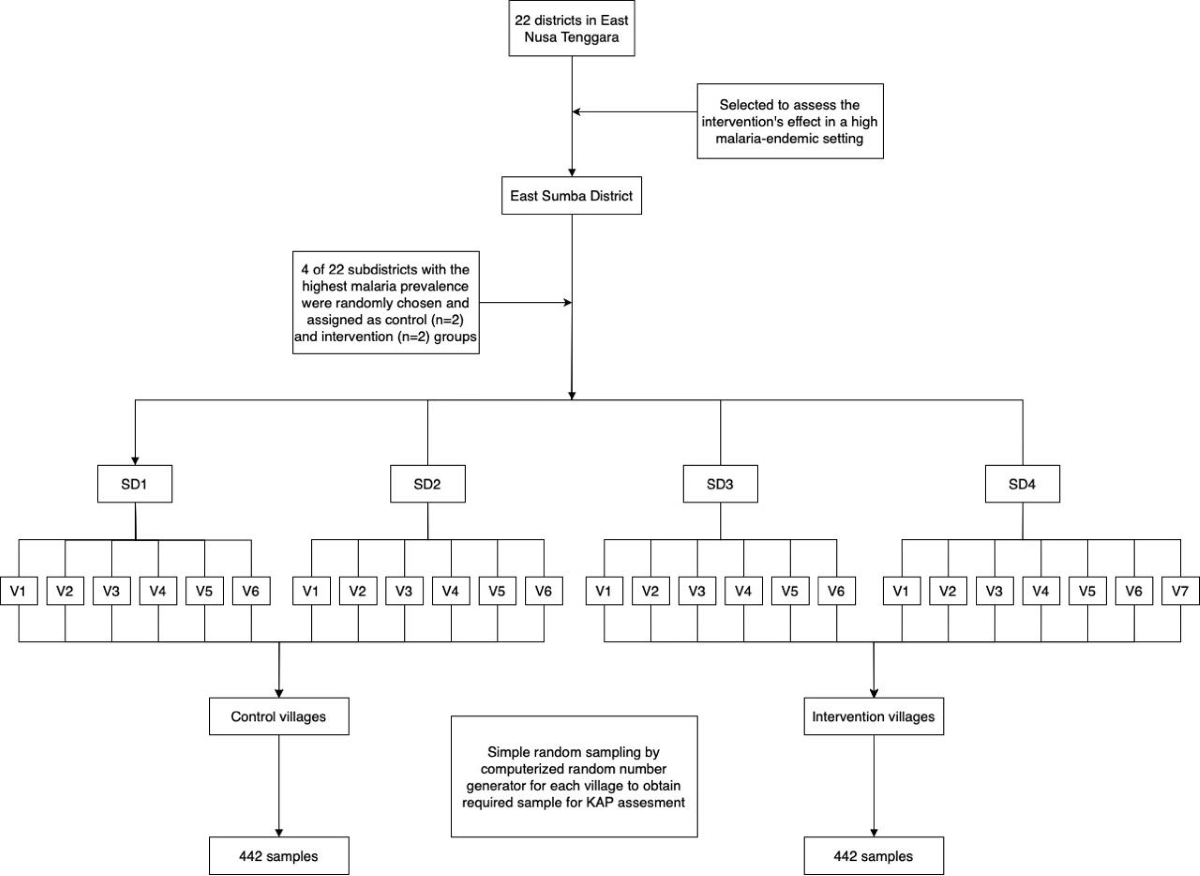
Flowchart of the selection of malaria-endemic settings and the village populations. KAP: knowledge, attitude, practice; SD: subdistrict; V: village.

This study used a cluster-assigned quasi-experimental design with pretest and posttest assessments in both control and intervention groups. The cluster unit for this study was the village, which was not randomized because the research team aimed to implement the intervention across all villages within a selected subdistrict, where the subdistrict leader would serve as a role model to encourage the community to improve malaria awareness. To obtain a representative sample, the research team randomly selected 4 of the 22 subdistricts, of which 2 were assigned as the intervention groups and 2 served as the control groups. The first 3 subdistricts each had 6 villages, and the fourth subdistrict had 7 villages; therefore, a total of 25 villages participated in the study, representing about 16% of all the villages in the district. Of these, 13 villages were assigned to the intervention group, while 12 villages formed the control group. The number of sampled households selected from each village was proportional to the number of households in that village. Within each selected village, households were randomly sampled to serve as an experimental unit as indicated in Figure 2.

### Sample Size Calculation

To determine the required sample size for the baseline prevalence of study outcomes, the research team applied the Leslie-Kish formula for calculating the sample size in cross-sectional studies [66]:




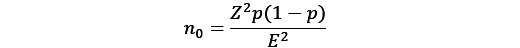




where n_0_ is the initial sample size; p is the prevalence of malaria awareness in ENT Province (48.8%) [39], Z is the confidence level at 95% (standard value of 1.96), and d is the relative precision (0.05); therefore, the initial sample size was 384 participants. For the population survey, a community correction factor of 2 was applied, doubling the required sample size to 768 participants. Additionally, a 15% buffer was added to account for potential incomplete data, primarily due to nonresponse or failure to complete interviews. Thus, the final sample size was determined to be 884 participants with 442 individuals in the control group and 442 individuals in the intervention group. This number served as a guideline for recruiting participants for the household survey, which was conducted both before and after the intervention.

### Eligibility Criteria

Individuals were eligible for recruitment if they had resided in the selected villages for at least 12 months and did not intend to migrate or relocate within the next 6 months after initial contact. Furthermore, participants were required to be at least 18 years old. All participants were required to provide written informed consent. For those who were unable to read or write, the consent form was read aloud by trained research assistants (RAs), and participants provided consent using a thumbprint on an ink pad. The RAs graduated from public health degrees and were local nurses with prior experience in data collection and employment at local public health centers. These professionals were recruited to ensure the study’s data collection process was conducted effectively.

### Study Procedure for Malaria Awareness Campaign

This study followed a 4-stage process to achieve its primary outcomes, as described in the following sections.

#### Phase I (Preparation Stage)

During this phase, the research team submitted an application to the Health Research Ethics Commission of Nusa Cendana University Kupang ENT Province, Indonesia, to obtain research ethics approval. Following approval, the research team coordinated with local authorities at various administrative levels, from the provincial level to the village level, in East Sumba District to obtain permission letters for conducting the study in the selected areas.

#### Phase II (Baseline Phase)

After securing the necessary approvals and obtaining the recommendation letter from the relevant authorities, the research team conducted a household survey with both the intervention and control groups. Before data collection, respondents were provided with a general overview of the study, and informed consent forms were obtained before their participation. The survey aimed to assess behavioral aspects of malaria and was conducted using a questionnaire that had been modified from a previous study conducted in rural ENT Province [67]. The questionnaire consisted of 40 questions designed to collect data on key malaria-related behaviors, including general knowledge of malaria, treatment-seeking behavior, and the use of malaria prevention methods. The questionnaire was completed by either the head of the household or another household member aged 18 years or older.

#### Phase III (Intervention Stage)

In the control group, malaria education for the local community was integrated into routine services at the local health center. These educational sessions were provided during the first prenatal visit by pregnant women to the health center, food supplement distribution activities, and immunization programs conducted during integrated service post activities at the village level. These activities were conducted once per month, and malaria-related information was disseminated once per village on average. The primary audience for these sessions included pregnant women, mothers with children younger than 5 years, and young children. Occasionally, malaria education was provided to all individuals attending the health centers. In the intervention group, malaria education followed the same structure as in the control group but was supplemented with a health education campaign targeting the entire village community.

The intervention followed the steps outlined in the following paragraphs.

First, the research team met with the subdistrict head and village leaders in the 2 selected subdistricts designated as the experimental areas. After obtaining their approval, the research team recorded a campaign message delivered by each subdistrict leader. The campaign content, developed by experts in malaria-related behavior, mathematical modeling, and rural development, included the main symptoms of malaria, prevention strategies, and the appropriate treatment-seeking behavior for suspected malaria cases. During the campaign, the subdistrict head occasionally emphasized key points in the local language to improve comprehension. The recorded message was then combined with a locally popular song to enhance engagement. The final recording was 15 minutes long. Second, 6 health promotion staff members from Baing and Mahu Public Health Centers were selected as RAs to play the recording. The recording was broadcast using loudspeakers to ensure wide community outreach. Third, the RAs played the recorded message once per week for 20 weeks in the 13 intervention villages. The intervention started in the fourth week of August 2024 and concluded in the second week of January 2025. In each selected village, the intervention was conducted in 4 different locations per week to ensure that all community members were reached. The principal investigator maintained weekly communication with the RAs to ensure that the intervention was being conducted properly. Over the 20-week intervention period, the research team monitored implementation by visiting the experimental villages once a month for 5 months.

#### Phase IV (Evaluation Stage)

At this stage, the research team conducted a household survey in both the intervention and control groups using the same questionnaire that was administered during the baseline phase of the study. The final data collection was done from the third week of January 2025 to the second week of February 2025. Since the research aims to assess the impact of the intervention on all rural adults, participants surveyed before and after the intervention may differ, depending on respondent availability during the data collection period.

### Data Collection Methods

Data were collected in face-to-face interviews, guided by a questionnaire adapted from a previously published study [67]. The questionnaire consists of 5 sections covering demographic characteristics, general knowledge of malaria, treatment-seeking behavior, practice of malaria prevention measures, and practice of malaria treatment-seeking behaviors. Each section contained questions to capture key behavioral aspects of malaria. A detailed list of the questions for each section is provided in Multimedia Appendix 1. Furthermore, this data collection was conducted by local nurses and health promotion workers from the selected health centers. These enumerators participated in a 1-day intensive training to improve their understanding of the study background and objectives. Moreover, enumerators were trained how to approach the potential participants in this study and how to conduct the interview following the guidance of this study and local culture. The same enumerators were responsible for recruiting participants during both baseline data collection and the postintervention phase. For secondary data collection, malaria register books from each public health center were reviewed to obtain data on the number of malaria cases and malaria-related deaths over a 10- year period from 2013 to 2023. Data on the number of malaria cases in each public health center throughout the district were collected from the Health Department of East Sumba District for the same period.

### Independent Variables of the Study

This study considers demographic and environmental factors as independent variables. Demographic variables include gender, age, education level, occupation, and socioeconomic status (SES). Respondents’ SES will be measured based on ownership of durable assets such as a television, refrigerator, car, electricity, telephone, and radio; the number of bedrooms; and home infrastructure conditions such as access to drinking water sources and the presence of water channels around the house [68]. Using principal component analysis, respondents’ SES will be categorized into 5 quantiles ranging from the lowest (quantile 1) to the highest (quantile 5) [69]. Environmental variables in the study include the location of the residence, categorized as rice fields, coastal areas, or hilly areas, and accessibility to villages, classified as easy, moderate, or difficult to access. Additionally, the study considered proximity to health facilities, including the type and distance of the nearest health facilities. For secondary data from the health center, patient records from 2013 to 2023 were reviewed for all patients with suspected malaria, and positive or negative cases were confirmed based on laboratory examination results. The number of malaria cases based on gender, age group, type of *Plasmodium*, time (year and month), location of residence, and type of treatment and the number of deaths since the period under review were collected from the 4 selected health centers.

### Outcome Variables

The study considers 10 potential outcomes variables, with the first 7 serving as the primary outcomes of the intervention, while the remaining 3 represent secondary findings. The first primary outcome of the study is improvement in the malaria awareness index among rural communities. If the malaria awareness index after the intervention is higher than that before the intervention, it indicates that the health education campaign effectively enhanced malaria awareness. This index will be assessed based on 12 questions covering general knowledge of malaria, treatment-seeking behavior, and knowledge of malaria prevention measures, following the methodology of a previous study [39]. Each correct response will be assigned a score of 1, while incorrect answers will receive a score of 0, with a total possible score of 12. Participants’ knowledge level will be categorized based on their accuracy rate: 0% (zero awareness), 1% to 59% (poor awareness), 60% to 79% (good awareness), and at least 80% (excellent awareness). Those classified as having excellent or good awareness will be considered aware of malaria, while participants in the other categories will be classified as unaware of malaria [39,70]. The malaria awareness index will be calculated before and after the intervention in both the intervention and control groups to evaluate the effectiveness of the health education campaign.

The second outcome variable in this study is improvement in AMTSB. This improvement will be demonstrated if the proportion of rural adults exhibiting AMTSB after the intervention is higher than before the intervention. AMTSB is defined as seeking malaria treatment at professional health facilities within 24 hours of experiencing malaria symptoms [41]. The proportion of rural adults engaging in AMTSB will be calculated both before and after the intervention in both the intervention and control groups. This assessment will be based on the 3 key questions from the malaria treatment-seeking behavior section of the questionnaire.

The third outcome variable in this study is improvement in good MPMK. This improvement will be demonstrated if the percentage of the rural population with good MPMK after the intervention is higher than before the intervention. A good level of MPMK is defined as the ability to correctly answer at least one-half of the total questions in the MPMK section, as indicated in previous studies [41,59]. The level of MPMK will be assessed before and after the intervention in both the intervention and control groups.

The fourth outcome variable is the use of bed nets during the dry season in high malaria-endemic settings. Community behaviors regarding bed net use may exhibit seasonal variations, as individuals may adjust their practices depending on weather conditions [71]. This outcome will be assessed based on the participants’ self-reported responses to a survey question asking whether they slept under a bed net the night before the survey.

The fifth outcome variable in this study is the variation in the use of a bed net in the dry and wet seasons. The literature indicates that bed net use might fluctuate based on seasonal weather conditions, as individuals adapt their malaria prevention behaviors accordingly [71,72]. This malaria awareness campaign began at the end of August 2024, during the dry season, and concluded in the second week of January 2025, which corresponds to the wet season in the study area. The study will examine seasonal differences in bed net use by analyzing responses to 4 key questions from the malaria prevention practice section of the questionnaire. The disparity in bed net use between dry and wet seasons will be assessed for both the intervention and control groups.

The sixth outcome variable in this study is improvement in good MPMP. Improvement will be demonstrated if the percentage of the rural population practicing good MPMP after the intervention is higher than before the intervention. A good level of MPMP is defined as the ability to correctly answer at least one-half of the total questions in the MPMP section, as indicated in a previous study [73,74].

The seventh outcome variable in this study is variation in access to basic drinking water and its associated factors among rural communities in ENT Province, Indonesia. Household access to basic drinking water is defined based on the WHO Joint Monitoring Program guidelines, which classify water sources into 4 categories: no service, unimproved, limited, and basic [75]. To analyze this variable, a dichotomous classification will be used, where households will be categorized as either having access to an improved drinking water source (Yes=1) or lacking access to an improved drinking water source (No=0). This classification will help determine disparities in access to safe drinking water and identify key factors influencing these differences.

The secondary outcomes of this study are derived from the analysis of patient medical records from 4 selected health centers, along with additional data from the Department of Health, East Sumba District. The eighth outcome variable focuses on the monthly number of malaria cases recorded over the past 10 years, from 2013 to 2023. By integrating data from both health centers and the Meteorological Agency of East Sumba, this study aims to explore the impact of climatic variables on malaria case fluctuations in this region, following the methodology of a previous study [76]. This analysis will provide valuable insights into seasonal and long-term trends in malaria transmission, which could help inform future malaria prevention and control strategies.

The ninth outcome variable in this study examines the demographic variations in malaria prevalence among communities residing in hilly and coastal areas over the past 10 years (2013-2023). Among the 4 selected health centers, Baing and Nggongi Public Health centers are located in coastal areas, whereas Mahu and Kananggar Public Health Centers are situated in hilly regions. Given that vector distribution and species composition may vary between different geographic settings [77], previous studies in Indonesia have reported significant differences in malaria prevalence between coastal and hilly areas [78]. By analyzing malaria case records from these health centers, this study aims to assess regional disparities in malaria burden and determine how demographic and environmental factors contribute to these differences. This information may help tailor malaria prevention and control efforts to the specific needs of different geographic regions.

The tenth outcome variable in this study investigates malaria prevalence and malaria-related mortality rates in East Sumba district over the past 10 years (2013-2023). Data on the number of malaria cases and malaria-related deaths were obtained from malaria register books at the health centers and malaria reports from the Health Department of East Sumba District. Therefore, the trends in malaria prevalence and mortality rates over the 10-year period in the study area will be revealed, following the methods of previous studies [79,80].

### Statistical Analysis

All statistical analyses will be conducted using SPSS version 27 (IBM Corp), and *P* values <.05 will be considered statistically significant.

For the first outcome variable, descriptive statistics will be used to summarize participants’ sociodemographic characteristics and environmental variables including gender, age group, SES, education level, family size, distance to the nearest health facilities, village accessibility, and household location. A chi-square test will be applied within each study group (intervention and control) to evaluate the association of basic malaria understanding, basic malaria knowledge, the level of malaria knowledge, and the level of malaria awareness before and after the intervention. Unpaired sample *t* tests will be used to investigate the statistical differences. Comparisons will be performed within groups (pre- and postintervention) and between groups to determine the impact of the intervention [61]. Furthermore, a binary logistic regression model will be used to identify the potential factors associated with malaria awareness in both the intervention and control groups [39].

For the second outcome variables, descriptive statistics will be used to summarize participants’ sociodemographic and environmental characteristics including gender, age group, SES, education level, family size, distance to the nearest health facilities, access to village, and household location for both study groups. A chi-square test will be used to evaluate the association between AMTSB and the sociodemographic and environmental characteristics of participants. Unpaired samples *t* tests will be used to assess the statistical differences. Comparisons will be conducted within and between groups for both pre- and postintervention periods. Moreover, binary logistic regression analysis will be used to identify predictors associated with AMTSB among participants in both study groups [41]. Only predictors that demonstrate statistically significant differences (*P*<.05) will be retained in the final model.

For the third variable response, participants’ sociodemographic characteristics and environmental variables in each group will be reported using descriptive statistics. The association between a good level of MPMK with predictors will be investigated using a chi-square test. Unpaired samples *t* tests will be used to investigate the statistical differences. Comparisons will be conducted within and between groups for both pre- and post-intervention periods. Furthermore, binary logistic regression analysis will be performed to identify key risk factors influencing MPMK [44,81] among rural communities in both study groups.

For the fourth outcome variable, descriptive statistics will be used to summarize participants’ sociodemographic characteristics and environmental variables based on the awareness that malaria can be prevented. Associations between bed net use and its predictors will be examined using a chi-square test. Binary logistic regression analysis will then be conducted to identify the primary risk factors associated with bed net use during the dry season among rural communities [71].

For the fifth outcome variable, participants’ sociodemographic characteristics, environmental variables, and malaria-related behavioral aspects in both the intervention and control groups will be analyzed. Data will be presented as proportions for each season (dry and wet seasons) to examine the seasonal variations in malaria prevention behaviors. The associations between bed net use and its predictors will be evaluated using a chi-square test for each season. The main risk factors for bed net use during the dry and wet seasons will be investigated using binary logistic regression analysis. The adjusted odds ratio will be used to quantify the strength of the associations between bed net use and its predictors in each season [71,72].

For the sixth outcome variable, participants’ sociodemographic characteristics, environmental variables, and behavioral aspects of malaria in each group will be reported using descriptive statistics. The association between a good level of MPMP and its predictors will be investigated using chi-square tests. Unpaired samples *t* tests will be used to investigate the statistical differences. Comparations will be performed within and between groups for both pre- and postintervention periods. Moreover, binary logistic regression analysis will be performed to identify the primary risk factors influencing MPMP [73,74] among rural communities in both study groups.

For the seventh outcome variables, descriptive statistics will be used to summarize participants’ sociodemographic characteristics, environmental variables, and malaria-related behaviors based on their source of drinking water. The associations between access to improved drinking water and its predictors will be assessed using chi-square tests. Furthermore, a binary logistic regression model will be used to determine predictors influencing access to improved drinking water [75].

For the eighth outcome variable, descriptive analyses of climatic factors across different subdistricts will be performed. Data will be aggregated at the subdistrict level, and statistical estimates will be generated for each variable. Specifically, the total number of malaria cases and the average malaria incidence will be computed. Climatic variables, including maximum, minimum, and mean temperature values, will be calculated. Moreover, data from all 22 subdistricts will be analyzed, focusing on malaria cases and climatic parameters, including maximum, minimum, and average temperature values. To depict the seasonal dynamics, seasonal variations in climatic parameters and malaria cases will be plotted, covering the 2 main seasons. A time series for both predictor and outcome variables will be plotted to deliver an overall perspective. A map of malaria cases will be generated. Scatterplots will be developed to illustrate relationships between malaria cases and predictor variables, following the methods of a previous study [76].

For the ninth outcome variable, malaria cases records from 2013 to 2023 will be analyzed across different geographic areas. Data from Baing and Nggongi Public Health Centers, representing coastal regions, will be combined, while records from Mahu and Kananggar Public Health Centers, representing hilly areas, will be aggregated separately. Malaria prevalence will be calculated on a monthly, yearly, and cumulative basis over the study period. To visualize the demographic trends, malaria prevalence will be plotted against key demographic variables. The disparity of malaria prevalence in demographic variables will be investigated using a chi-square test [10,82].

For the tenth outcome variable, descriptive statistics will be used to calculate frequencies and percentages of overall malaria prevalence and trends in malaria transmission considering variations by season, month, year, sex, age, and species of malaria parasite. Epidemiological characteristics, including age group, sex, and *Plasmodium* species–adjusted malaria-related mortality rates in East Sumba District will be presented as percentages followed by their 95% confidence intervals. Chi-square tests will be used to compare the associations of malaria burden by age groups and sex. The malaria prevalence and mortality rate will be illustrated in a map to assess the distributions of the cases geographically [79,80].

### Ethical Considerations

We adhered to the Declaration of Helsinki to ensure that the rights, integrity, and confidentiality of the respondents are strictly protected. All respondents signed consent forms before being interviewed. For this interventional study, we received human ethics approval from the Human Ethics Committee of Nusa Cendana University Kupang ENT Province Indonesia (ethics ID: 42/UN15.21/KEPK/2024) as shown in Multimedia Appendix 2. All respondents who participated in this study were involved voluntarily without compensation in the form of material; however, they received additional knowledge about malaria. Data obtained from this study will be strictly kept by the principal investigator, and no personal respondent information will be shared during the dissemination of the study results. The research team secured official permission letters from relevant authorities at the provincial, district, subdistrict, and village levels to conduct the study within the designated areas. Written consent forms were collected from all participants participating in this study.

### Dissemination

The findings of this interventional study will be disseminated through national and international conferences and published in peer-reviewed journals.

## Results

The time frame of the project is presented in Table 1.

**Table 1:** Timeline of research phases.

Research phases	January-May 2024	June-July 2024	August 2024-January 2025	February 2025	March 2025-December 2026
Questionnaire and question guide development	✓	—^a^	—	—	—
Ethics approval	—	✓	—	—	—
Training of field workers	—	✓	—	—	—
Household surveys for pretest data	—	—	✓	—	—
Data entry	—	—	✓	—	—
Data analysis	—	—	✓	—	—
Manuscript preparation and submission	—	—	✓	—	—
Intervention in 13 villages	—	—	✓	—	—
Household surveys for posttest data	—	—	—	✓	—
Data entry	—	—	—	—	✓
Data analysis for the final outcome	—	—	—	—	✓
Manuscript preparation and submission	—	—	—	—	✓

^a^Not applicable.

Primary data collection for the baseline survey was conducted between August 2024 and September 2024. A total of 894 rural adults participated in face-to-face interviews in both the intervention and control groups. These participants were drawn from 25 villages across East Sumba District. The intervention commenced in the last week of August 2024 and concluded in the second week of January 2025. The project is currently in the process of drafting multiple research papers, which will be submitted for publication in peer-reviewed journals to disseminate the findings. This study was funded by the Directorate General of Higher Education, Ministry of Education and Culture of the Republic of Indonesia (main contract number: 073/E5/PG.02.00.PL/2024) on June 11, 2024, and an extension contract number from the Research Center of Nusa Cendana University Kupang ENT Province (contract number: 448/UN15.22/SP2H/PL/2024) on June 12, 2024.

## Discussion

### Principal Findings

This research endeavors to examine the impact of integrating multiple media into an interventional study designed to influence malaria-related behavioral changes among rural communities. The intervention incorporated local governmental leaders, traditional music, and loudspeaker announcements, recognizing their potential to enhance community engagement and facilitate effective malaria awareness campaigns. Local authorities play a pivotal role in public health initiatives as they serve as trusted figures within their communities and possess extensive experience in mobilizing the local population [63,83]. Furthermore, music has been shown to be a powerful tool for fostering social connection and disseminating health information, particularly in efforts to promote preventive behaviors for infectious diseases [62]. The use of loudspeakers as a communication medium has also been identified as an effective approach for delivering malaria health messages and ensuring continuous information dissemination in rural settings [61]. Moreover, integrating music-based education enhances learning outcomes among populations with low literacy rates [84], making it a particularly effective strategy for engaging rural communities. This is particularly relevant in ENT Province, where high school dropout rates remain a challenge [85,86]. By leveraging these culturally relevant and widely accessible communication methods, this quasi-experimental study has the potential to generate a significant impact on improving the malaria awareness index, AMTSB, knowledge of malaria prevention measures, and practice of malaria prevention measures among rural adults in ENT Province, Indonesia.

Interventional studies have demonstrated a positive impact on improving malaria-related behaviors at the community level across various settings [50,51,55,60,87-89]. In SEA, several interventional studies have shown significant contributions to behavioral change in malaria prevention and treatment [52,61,90,91]. For instance, a study conducted in Northern Myanmar found that loudspeaker announcements delivering malaria prevention and treatment messages over 6 months significantly increased malaria awareness among villagers [61]. Similarly, a school-based educational intervention in India demonstrated a substantial improvement in malaria prevention and control knowledge among students in grades 9 to 12 [90]. In Pakistan, an interventional study targeting pregnant women indicated that the use of LLINs increased significantly following a 12-week health education program [52]. In Bhutan, a community-directed educational intervention on malaria prevention, implemented over 6 months, led to improvements in malaria-related knowledge, attitudes, and prevention practices among rural communities in Sarpang district [91]. In Indonesia, there is a notable gap in interventional studies focused on malaria education. Most existing research has been conducted in school settings [60] and is primarily concentrated in the western region of the country [59]. To the best of our knowledge, this is the first study in SEA to investigate the combined impact of local leadership, traditional music, and loudspeaker announcements as intervention media to enhance malaria knowledge, improve prevention practices, and encourage early treatment-seeking behaviors among rural communities affected by malaria.

The data collection instruments used in this study were adapted from a validated questionnaire, with modifications made to ensure ease of administration. The measurement of key variables, including the malaria awareness index, AMTSB, MPMK, and MPMP, was based on standardized indicators from reputable international journals. This methodological approach allows for comparability with national and international studies, enhancing the credibility and applicability of the findings. Consequently, the study’s results will serve as an important reference for guiding malaria health policy in rural Indonesia and supporting the national commitment to achieving malaria elimination by 2030.

Despite its strengths, this study has several limitations. First, village selection was not randomized for the control and intervention groups, which may introduce potential selection bias. However, the allocation strategy ensured that the distribution of villages between study groups was comparable in terms of malaria endemicity, and the intervention was designed to maximize its impact by including all villages within a selected subdistrict. Second, most of the data collected in this interventional study relied on self-reported responses from participants, covering aspects such as general malaria knowledge, symptoms, diagnosis, and prevention measure knowledge. This reliance on self-reported data introduces the potential for recall bias. However, to mitigate this limitation, all MPMP were directly observed by RAs during data collection, improving the reliability of reported behavioral measures.

### Conclusions

This study is expected to provide significant findings to comprehensively investigate the change in behavioral aspects of malaria among rural communities following the implementation of a local wisdom-based malaria intervention. Changes in the malaria awareness index, MPMK, MPMP, disparity in using core prevention measures such as LLINs in 2 different seasons, and malaria treatment–seeking behaviors among rural communities due to the implementation of a local wisdom-based health education campaign will be recognized. These findings will provide evidence-based insights into the effectiveness of locally driven health education strategies in malaria-endemic regions. Therefore, this research will contribute to the development of contextually relevant malaria policies in Indonesia. Furthermore, it aligns with global malaria elimination efforts, supporting Indonesia’s commitment to achieving a malaria-free status by 2030.

## Appendix

Multimedia Appendix 1 The questionnaire for primary data collection in pre and post-test.

Multimedia Appendix 2 Ethics approval of nusa cendana university kupang east nusa tenggara indonesia.

## Abbreviations

AMTSB: appropriate malaria treatment-seeking behavior
ENT: East Nusa Tenggara
LLIN: long-lasting insecticide-treated bed net
MPMK: malaria prevention measures knowledge
MPMP: malaria prevention measures practice
RA: research assistant
SEA: Southeast Asia
SES: socioeconomic status
WHO: World Health Organization
