# Supplementary material for: Health Education Campaign to Improve Malaria Knowledge, Prevention, and Treatment Behaviors in Rural East Nusa Tenggara Province, Indonesia: Protocol for a Cluster-Assigned Quasi-Experimental Study
Source: JMIR Res Protoc. 2025 May 1;14:e66982. doi: 10.2196/66982 (PMC12082057; doi:10.2196/66982)
Supplement: Multimedia Appendix 1 [file resprot_v14i1e66982_app1.pdf]

QUESTIONNAIRE

Date of interview : ________/_________/ _____

Name of interviewer : _________________________

Mobile number : _________________________

Participant Info

ID Number : __________________________

Address : __________________________

Hamlet /Village : ____________/____________

Category of Village : **1 = Easy access** **2 = Moderate access** **3 = Difficult access**

Sub-District : _________________________

Geographical conditions : **1 = Coastal area** **2 = Rice fields** **3 = Hills**

4 = Forest **5 = Swamps**

SECTION 1: DEMOGRAPHIC CHARACTERISTICS		
**Questions**	**Coding Categories**	**Answer Code**
1.1 How old are you?	……. years (age in current year)	
1.2 What is your gender?	1 = Male 2 = Female	
1.3 What is your highest level of education?	1 = No education<br>2 = Primary school<br>3 = Junior High school<br>4 = Senior High School<br>5 = Diploma<br>6 = Bachelor or above	
1.4 What is your main occupation?	1= Farmer<br>2 = Housewife<br>3= Fisherman<br>4 = Labour<br>5 = Trader/Merchandiser<br>6 = Student<br>7 = Unemployed<br>8 = unable to work<br>9 = Govt. or non-govt. employment (Officers/ Police/Army, teachers)<br>10 = others (Please specify):	
1.5 What is your monthly income (approximately)	Indonesian currency :	
1.6 What is your household monthly income (approximately)	Indonesian currency :	
1.7 What is the main material of the floor in your house?	1 = Ceramics<br>2 = Plastering cement<br>3 = Board<br>4 = Soil<br>5 = Others (Please specify) :	
1.8 What is the main material of the wall of your house?	1 = Plastering cement<br>2 = Zinc<br>3 = Bamboo /bamboo webbing/ rattan	

	4 = Wood/board/plywood<br>5 = Others (Please specify):	
1.9 What is the main material of the ceiling of your house?	1 = Concrete<br>2 = Wood/Plywood<br>3 = Bamboo webbing<br>4 = None<br>5 = Others (Please specify):	
1.10 From the following household items, how many items do you have? Number = ………	1 = Radio 2 = TV 3 = TV cable<br>4 = Bike 5 = Motorbike<br>6 = Car 7 = Mobile phone.<br>8 = Electricity 9 = Fridge<br>10 = AC 11 = Tractor 12 = Generator	
1.11 What is the main source of drinking water for your family?	1 = Running tap water in dwelling<br>2 = Running tap water of public<br>3 = Drilled well pump<br>4 = Well<br>5 = Spring<br>6 = River<br>7 = Irrigation<br>8 = Other (Please specify)	
1.12 What is the nearest health service of your residence?	1 = Public Health centres<br>2 = Subsidiary Public Health centres<br>3 = Village health Post<br>4 = Midwife practices<br>5 = Village maternity posts<br>6 = Polyclinic<br>7 = General practice<br>8 = Other (Please specify):	
1.13 What is the distance to the nearest health facility?	……. (Distance in Km)	

**SECTION 2: GENERAL KNOWLEDGE ABOUT MALARIA**		
2.1 Have you heard about malaria before?	1=Yes 2 = No	
If you said yes to question 2.1, please complete question 2.2 to 2.13. Otherwise go to section 3		
2.2 Where did you hear about malaria?	1 = TV<br>2 = Radio<br>3 = Newspaper<br>4 = Friend/Family member<br>5 = Local health facility<br>6 = School<br>7 = Church<br>8 = Mosque<br>9 = Community meetings<br>10 = Pamphlets<br>11 = Other (Please specify) :	
2.3 Do you know some symptoms of malaria?	1 = Yes 2 = No	
2.4 What do you think are the main symptoms of malaria infection?	1=Fever 2 = Shivering<br>3= Sweating 4 = Headache<br>5=Nausea and vomiting 6 = Diarrhoea<br>7=Dizziness 8 = Loss of appetite<br>9=Body weakness 10 = Joint pains<br>11 = Other (Please specify)	
2.5 In your opinion, what is the main causes of malaria?	1 = Mosquito bite<br>2 = Witchcraft<br>3 = Drinking dirty water<br>4 = Cold weather<br>5 = Unhygienic environment<br>6 = Close contact with a malaria sufferer	

	7 = Other (Please specify):	
2.6 Do you think malaria can be prevented?	1 = Yes 2 = No	
2.7 What personal protective measures can you use to guard against malaria infection?	1 = Sleeping with non-insecticide treated net<br>2 = Sleeping with insecticide treated net received <=3 year ago<br>3 = Sleeping with insecticide treated net received > 3 year ago<br>4 = Using mosquito coil<br>5 = Keep house clean<br>6 = Covering ventilation with anti-mosquito nets<br>7 = Using Indoor Residual Spraying<br>8 = Wearing long sleeved clothes when go outdoors at night<br>9 = Taking anti-malaria drugs when staying at malaria endemic areas.<br>10 = Other (Please specify) :	

**SECTION 3: TREATMENT SEEKING BEHAVIOUR**		
3.1 If you or member of your family present with the signs and symptoms of malaria would you/they seek treatment?	1 = Yes<br>2 = No	
3.2 If yes to 3.1, how soon after suspecting that you or your family member are infected with malaria would you/they seek treatment?	1 = One day (Within 24 hours)<br>2 = 2 days<br>3 = 3 days<br>4 = 4 days or more<br>5 = I did not go for treatment	
3.3 If yes to 3.1 and you or member of your family present with the signs and symptoms of malaria where would you/they seek treatment?	1 = Public health facilities<br>2 = Private health facilities<br>3 = Traditional healer<br>4 = Pharmacy<br>5 = self-treat<br>6 = other (Please specify):	

**SECTION 4: PRACTICE OF PERSONAL PROTECTION**		
4.1 Does anyone in the household use any personal protective items to help prevent malaria infection?	1 = Yes 2 = No	
4.2 What personal protective measures do you use to guard against malaria infection?	1 = Sleeping with non-insecticide treated net<br>2 = Sleeping with insecticide treated net received <=3 year ago<br>3 = Sleeping with insecticide treated net received > 3 year ago<br>4 = Using mosquito coil/electric anti mosquito mats<br>5 = Keep house clean<br>6 = Covering ventilation with anti-mosquito nets<br>7 = Using Indoor Residual Spraying<br>8 = wearing long sleeved clothes when go outdoors at night<br>9 = Taking anti-malaria drugs when staying at malaria endemic areas.<br>10 = Other (Please specify) :	
4.3 Do you have any mosquito net?	1 = Yes 2 = No	

4.4 What type of mosquito net do you have?	1 = Long Lasting Insecticide-Treated Bet Nets (LLINs)<br>2 = Non LLINs<br>3 = LLINs and Non LLINs	
**4.5 If you choose option 1 to question 4.4,**<br>**a. How many mosquito nets do you have?**<br>**b. When did you get this bed net:**<br>**c. Does this bed net eligible to be used?**<br>**d. How many sleeping groups do you have?**	 ……… (Number of LLINs)<br>Year ……<br>1 = Yes 2 = No<br>………….. (Number of sleeping groups)	
**4.6 If you choose option 2 to question 4.4,**<br>**a. How many mosquito nets do you have?**<br>**b. Does this bed net eligible to be used?**	 …………… (Number of non-LLINs)<br>1 = Yes 2 = No	
If you choose option 3 to question 4.4, please complete questions 4.5 and 4.6		
4.7 a. Did you sleep under bed net last night?<br>***b. If yes to question 4.7a, What type of bed net do you use?***	1 = Yes 2 = No<br>1= LLINs 2 = Non LLINs	
4.8 Did all of your family members sleep under mosquito net last night?	1 = Yes 2 = No	
4.9 What is your family size?	……	
4.10 If you said no to question 4.7a, what is the main reason?	1 = Too hot<br>2 = Bed net was not hung up<br>3 = Bed net was not in good condition /torn<br>4 = Mosquito net is for children<br>5 = Mosquito net is for pregnant women<br>6 = No mosquitoes<br>7 = Not get used to<br>8 = other (Please specify):	

**SECTION 5 : PRACTICE FOR MALARIA TREATMENT**		
5.1 Have you ever suffered from malaria?	1 = Yes 2 = No	
If you answer yes on question 5.1, please do question 5.2 to 5.11, otherwise go to section 6		
5.2 When did it occur?	Month: …….. Year: …….	
5.3 What symptoms did you have then?	1 = Fever 2 = Shivering<br>3 = Sweating 4 = Headache<br>5 = Others (Please specify):	
5.4 How soon after suffering from malaria did you seek treatment?	1 = One day (Within 24 hours)<br>2 = 2 days<br>3 = 3 days<br>4= 4 days or more<br>5 = I did not go for treatment	
5.5 If you sought treatment, where did you go to seek treatment?	1 = Public health facilities<br>2 = Private health facilities<br>3 = Pharmacy<br>4 = Traditional healer<br>5 = self-treatment<br>6 = other (Please specify):	
5.6 If your response to 5.5 was option 1 or 2 or 3 , did you receive blood test?	1 = Yes 2 = No	
5.7 If your response to 5.4 was option 1 or 2 or 3, did you receive any medicine?	1 = Yes 2 = No	
5.8 If you responded yes to 5.6, did you receive the medicine stated below?		

5.8.1 Artemisinin-based Combination Treatment (ACT) + 1 day Primaquine<br>***5.8.2 Artemisinin-based Combination Treatment (ACT) + 14 days Primaquine***<br>***5.8.3 Other medicine (Please specify):***	1 = Yes 2 = No<br><br>1 = Yes 2 = No	
5.9 Did you consume the medicine in question 5.7 until finish?	1 = Yes 2 = No	
5.10 If you answered no to question 5.8, what was the main reason for that?	1 = Forgot to take<br>2 = Felt better<br>3 = Not comfortable due to drug's side effects<br>4 = Other (Please specify) :	

THANK YOU VERY MUCH

We have to come to the end of our interview. Your time, honest opinions and valuable contributions are highly appreciated. Do you have any questions?
