# Supplementary material for: Health Education Campaign to Improve Malaria Knowledge, Prevention, and Treatment Behaviors in Rural East Nusa Tenggara Province, Indonesia: Protocol for a Cluster-Assigned Quasi-Experimental Study
Source: JMIR Res Protoc. 2025 May 1;14:e66982. doi: 10.2196/66982 (PMC12082057; doi:10.2196/66982)
Supplement: Multimedia Appendix 2 [file resprot_v14i1e66982_app2.pdf]

KEMENTERIAN PENDIDIKAN, KEBUDAYAAN,
RISET DAN TEKNOLOGI
UNIVERSITAS NUSA CENDANA
FAKULTAS KEDOKTERAN DAN KEDOKTERAN HEWAN
KOMISI ETIK PENELITIAN KESEHATAN

Sekretariat : Lantai 2 Gedung Laboratorium Terpadu
Alamat : Jl. Adisucipto Penfui, PO. BOX 104, Kupang 85001, NTT
Telepon/Fax : (0380) 881972
Email : info.lkkh@undana.ac.id

REKOMENDASI PERSETUJUAN ETIK
Nomor : 42/UN15.21/KEPK/2024

Komisi Etik Penelitian Kesehatan Fakultas Kedokteran dan Kedokteran Hewan Universitas Nusa Cendana, setelah melalui pembahasan dan penilaian, pada rapat tertanggal 22 Juli 2024 telah memutuskan, protokol penelitian berjudul :

"Pengaruh Promosi Kesehatan Berbasis Kearifan Lokal Terhadap Peningkatan Kesadaran Malaria Masyarakat Pedesaan Di Kabupaten Sumba Timur"

dengan Peneliti Utama : Robertus Dole Guntur, S.Si., M.Maths.Sc, PhD

No. Register

U	N	0	2	2	4	0	6	4	0

yang diterima pada tanggal : 20 - 06 - 2024
Perbaikan diterima tanggal : -

Dapat disetujui untuk dilaksanakan di **4 Kecamatan dan 4 Puskesmas di Kabupaten Sumba Timur yaitu Kecamatan Mahu, Kecamatan Wula Waejelu, Kecamatan Karera, Kecamatan Paberiwai, Puskesmas Mahu, Puskesmas Baing, Puskesmas Kanangar dan Puskesmas Karera.** Persetujuan Etik ini berlaku sejak tanggal ditetapkan sampai dengan batas waktu pelaksanaan penelitian seperti tertera dalam protokol.

Pada akhir penelitian, peneliti **harus menyerahkan laporan perkembangan dan laporan akhir penelitian** kepada KEPK Fakultas Kedokteran dan Kedokteran Hewan Undana. Jika ada perubahan protokol dan/atau perpanjangan penelitian, harus mengajukan kembali permohonan kajian etik penelitian.

Kupang, 22 Juli 2024

Komisi Etik Penelitian Kesehatan Fakultas Kedokteran dan Kedokteran Hewan Undana

Ketua

dr. Teguh Dwi Nugroho, M. Biomed.,Sp.B
NIP. 19840502 202321 1 019

Sekretaris I,

dr. Desi Indria Rini, M.Biomed
NIP. 19800130 200801 2 015

Dipindai dengan CamScanner

MINISTRY OF EDUCATION, CULTURE, RESEARCH, AND TECHNOLOGY
NUSA CENDANA UNIVERSITY
FACULTY OF MEDICINE AND VETERINARY MEDICINE
HEALTH RESEARCH ETHICS COMMITTEE
Secretariat: 2nd Floor, Integrated Laboratory Building
Address: Jl. Adisucipto Penfui, PO. BOX 104, Kupang 85001, East Nusa Tenggara, Indonesia
Telephone/Fax: (0380) 881972
Email: info_fkkh@undana.ac.id

ETHICAL APPROVAL RECOMMENDATION

Number: 42/UN15.21/KEPK/2024

The Health Research Ethics Committee of the Faculty of Medicine and Veterinary Medicine at Nusa Cendana University, after deliberation and assessment during a meeting on July 22, 2024, has decided on the following research protocol titled:

"The Impact of Local Wisdom-Based Health Promotion on Increasing Malaria Awareness Among Rural Communities in East Sumba Regency"

Principal Investigator: Robertus Dole Guntur S.Si. M.Maths.Sc PhD

Registration Number: UN02240640

Received on : June 20, 2024
Revisions received on : -

It is approved to be carried out in **4 sub-districts and 4 Public Health Centres in East Sumba Regency, specifically in Mahu Sub-District, Wula Waejelu Sub-District, Karera Sub-District, Paberiwai Sub-District, as well as Mahu Public Health Center, Baing Public Health Center, Kanangar Public Health Center, and Karera Public Health Center**. This Ethical Approval is valid from the date of issuance until the completion of the research, as indicated in the protocol.

At the end of the research, the researcher is **required to submit progress reports and a final research report** to the Ethics Committee of the Faculty of Medicine and Veterinary Medicine, Nusa Cendana University. If there are any protocol changes and/or research extensions, a new ethics review request must be submitted.

Kupang, July 22, 2024

Health Research Ethics Committee of the Faculty of Medicine and Veterinary Medicine, Nusa Cendana University

**Chairman:**	**Secretary I:**
dr. Teguh Dwi Nugroho, M. Biomed.,Sp.B	dr. Desi Indira Rini, M. Biomed
NIP. 19840502 202321 1 019	NIP. 19800130 200801 2 015
